# 

**Published:** 1884-12

**Authors:** 


### CONTENTS.

ORIGINAL COMMUNICATIONS: PAGE.

I. Recent Cases in Surgical Practice. By Edmund Andrews, M. D., LL. D., Professor of Clinical Surgery. Chicago Medical College, Medical Department, Northwestern University. Read Before the Chicago Medical Society, Nov. 10, 1884 ... 581

II. The Muriate of Cocaine—A Clinical Lecture, by Dr. E. L. Holmes, at the Illinois Eye and Ear Infirmary ... 489

III. Albuminuria Associated with Habitual Use of Opium. By F. P. Mann, M. D. ... 492

IV. A Case of Separation of the Symphysis Pubis during Labor. By E. F. Eldridge, M. D... 495

EDITORIAL:

The Iodoform, Intra-uterine Treatment During the Puerperium ... 496

A "No-Cure, No-Pay" Decision ... 498

BOOK REVIEWS:

Text Book of Medical Jurisprudence and Toxicology. By John J. Reese, M. D., Professor of Medical Jurisprudence and Toxicology in the University of Pennsylvania. 8vo., pp. xii., 606. Philadelphia: P. Blakiston, Son & Co., 1884. ... 499

A Practical Treatise on Fractures and Dislocations. By Frank Hastings Hamilton, A. B., A. M., M.D., LL. D. Late Professor of Surgery in Bellevue Hospital Medical College, and Surgeon to Bellevue Hospital, New York. Seventh American Edition, Revised and Improved. Octavo. pp. xxxi, 1005. Philadelphia: Henry C. Lea's Son & Co. 1884... 500

A Manual of Physiology: A Text-Book for Students. By Gerald F. Yoe, M. D., F. R. C. S. Professor of Physiology in King's College, London. Octavo, pp. xxiv., 749. Philadelphia: P. Blakiston, Son & Co. 1884... 500

Allen's Human Anatomy: Section V. Organs of Sense, Organs of Digestion, and Genito-Urinary Organs. By Harrison Allen, M. D., Professor of Physiology in the University of Pennsylvania. Philadelphia: Henry C. Lea's Son & Co. 1883... 501

DOMESTIC CORRESPONDENCE:

County Insane Asylum Affairs. The following communications explain themselves: An Open Letter to the Commissioners of Cook County, Illinois... 502

The Perpetual Bath... 507

FOREIGN CORRESPONDENCE:

Letter from Darmstadt... 513

From the Medical Clinics of Prof. Reigel, at Giessen, for Advancement in the Diagnosis of Acute Miliary Tuberculosis. By Dr. Proebsting, in Lippstringe, formerly Clinical Assistant... 516

SELECTIONS:

External Use of Chloroform in Labor. By A. Svanberg, M. D., Sweden... 526

SOCIETY PROCEEDINGS:

Chicago Gynecological Society... 529

Chicago Society of Ophthalmology... 541

Chicago Pathological Society... 547

Chicago Medical Society... 567

BOOKS RECEIVED... 575

